# Impacts of COVID-19 pandemic prevention measures to the palliative care in Taiwan

**DOI:** 10.3389/fpubh.2024.1411185

**Published:** 2024-07-25

**Authors:** Meng-Ping Wu, Sheng-huang Hsiaog, Tsun-Cheng Huang, Da-Chen Chu, Chieh-Yu Liu

**Affiliations:** ^1^Department of Nursing, Taipei City Hospital, Taipei City, Taiwan; ^2^College of Nursing, National Taipei University of Nursing and Health Sciences, Taipei City, Taiwan; ^3^Department of Psychology and Counseling, University of Taipei, Taipei City, Taiwan; ^4^Superintendent Office, Taipei City Hospital, Taipei City, Taiwan; ^5^Administrative Deputy Superintendent Office, Taipei City Hospital, Taipei City, Taiwan; ^6^Taipei City Hospital, Taipei City, Taiwan; ^7^Department of Health Care Management, National Taipei University of Nursing and Health Sciences, Taipei City, Taiwan; ^8^Department of Research and Development, Taipei City Hospital, Taipei City, Taiwan

**Keywords:** COVID-19, palliative care, discharge planning, end-of-life, advance care planning

## Abstract

**Background:**

Prevention measures for palliative care and the provision of discharge planning services for inpatients in Taiwan before and during the COVID-19 pandemic had not been investigated. This study was aimed to investigate the factors associated with heightened palliative care needs and increased mortality rates.

**Methods:**

This research adopts a retrospective case–control study design. The investigation encompasses patients admitted before the pandemic (from January 1, 2019, to May 31, 2019) and during the COVID-19 pandemic (from January 1, 2020, to May 31, 2020). The case group consisted of 231 end-of-life inpatients during the pandemic, control group was composed of the pool of inpatients with pre-pandemic and matched with cases by sex and age in a 1:1 ratio.

**Results:**

The results showed that the prevalence of respiratory failure symptoms (*p* = 0.004), residing in long-term care facilities (*p* = 0.017), palliative care needs assessment scores (*p* = 0.010), as well as the provision of guidance for nasogastric tube feeding (*p* = 0.002), steam inhalation (*p* = 0.003), turning and positioning (*p* < 0.001), percussion (*p* < 0.001), passive range of motion (*p* < 0.001), and blood pressure measurement (*p* < 0.001). Furthermore, the assessment of the necessity for assistive devices, including hospital beds, also exhibited statistically significant variations (*p* < 0.001). Further investigation of the factors associated with high palliative care needs and the risk of mortality for both the case and control groups. Risk factors for high palliative care needs encompassed assessments of daily activities of living, the presence of pressure ulcers, and the receipt of guidance for ambulation. Risk factors for mortality encompassed age, a diagnosis of cancer, palliative care needs assessment scores, and the provision of guidance for disease awareness.

**Conclusion:**

This research highlights the heightened risk of COVID-19 infection among end-of-life inpatients during the COVID-19 pandemic. The findings of this study may advance care planning to alleviate avoidable suffering. To meet the needs of inpatients during pandemic, healthcare professionals should undergo comprehensive palliative care training and receive policy support.

## 1 Introduction

Taiwan experienced a quite different COVID-19 outbreak course from Asia countries. When the first confirmed COVID-19 case was reported at the end of 2019 at Wuhan in China, and Taiwan Center for Disease Control (CDC) officially announced warning at Feb 20, 2020. From Feb 2020 to April 2021, Taiwan CDC adopted a series of prevention measures, including purchasing of masks by real name, prohibiting inbound tourists from serious COVID-19 pandemic countries,…etc. (1). However, due to lack of COVID-19 vaccines, Taiwan eventually encountered serious outbreak of COVID-19 from May 2021 till the end of 2022 (2). During the COVID-19 pandemic, according to data from Taiwan’s Central Epidemic Command Center, at the end of June 2022, there were over 500 million confirmed cases of COVID-19 worldwide. Taiwan reported 4,362,227 confirmed cases with 8,392 fatalities. While the majority of COVID-19 patients in Taiwan experienced mild or asymptomatic symptoms, there were still severe cases requiring oxygen therapy and admission to intensive care units. These severe cases could be complicated by conditions such as acute respiratory distress syndrome (ARDS), sepsis, septic shock, multiple organ failure, or cognitive impairment (3, 4).

Due to the delayed outbreak of COVID-19 pandemic in Taiwan, when inpatients in the end-of-life stage require palliative care, effective communication with their families becomes crucial, because they need to experience longer worry than before. Additionally, close collaboration among the healthcare team is essential. The implementation of infection control measures, the need to reduce contact and the resulting constraints on healthcare services, combined with the overwhelming workload of healthcare personnel, have made the provision of palliative care increasingly challenging (5, 6). Therefore, it is advisable to prioritize the early provision of palliative care based on patient and family needs before the onset of severe illness. This approach involves: (1) Avoiding the provision of intensive life-sustaining treatments against the patient’s wishes; (2) Preventing unnecessary high-intensity care when there is a strain on healthcare capacity; (3) To ensure that patients do not lose the ability to make decisions about unwanted care when their condition deteriorates, advance care planning (ACP) should be implemented proactively (5, 7). Furthermore, when clinicians address the topic of end-of-life decisions, they should engage in immediate communication with patients and their families regarding informed consent for Cardiopulmonary Resuscitation (CPR). This process involves: (1) Assessing the patient’s values and treatment goals, such as options for unnecessary life extension or considerations related to quality of life; (2) Explaining and discussing how, when, and why CPR is performed, providing a comprehensive overview of the pros and cons, including concerns about potentially not aligning with the patient’s care goals; (3) Offering a clear statement of non-consent for CPR from the patient or their family, while evaluating their understanding of this consent statement. Therefore, especially in the context of the COVID-19 pandemic, it becomes imperative to ensure that clinicians facilitate high-quality ACP and discussions about care goals to ascertain palliative care needs (8).

To enhance the quality of care for inpatients during the COVID-19 pandemic, it is essential to identify their care needs. Upon admission, the following assessments should be conducted: (1) home care; (2) rehabilitation therapy; (3) referrals to community resources; (4) assistive device assessments and utilization; (5) transitional services; (6) life reconstruction; (7) psychological counseling; (8) palliative care needs. These assessments should be part of comprehensive discharge planning services (8), ensuring the crucial processes for discharge planning (9). Discharge planning services constitute a process rather than a singular event, necessitating tailored, ongoing care planning discussions and coordination with interdisciplinary teams, patients, and their families. This involves the development of individualized care plans (8, 10). Through discharge planning services, it is ensured that patients, upon discharge, have the necessary physical stability, adequate support, psychological resilience, and sufficient information and knowledge (8, 11). It is important to enhance their non-functional health literacy during the COVID-19 pandemic (12).

The lack of integration between public health policies and clinical care is a challenge faced by most healthcare systems worldwide. This challenge has become even more pronounced, especially in the context of the COVID-19 pandemic (13). The absence of cross-agency coordination and comprehensive decision-making hampers the flexibility and capacity to respond effectively to the pandemic (14). In the face of the COVID-19 crisis, it is essential to foster mutual trust and collaboration within healthcare teams, delegation in leadership, ensure accountability among frontline personnel, encourage healthcare professionals, patients, and their families to demonstrate altruism, and promote empathy, mutual assistance, and selfless dedication among the general public (15, 16). This approach aims to ensure that during the critical phases of the pandemic, both the reduction of risks for patients, families, and healthcare personnel and the continued provision of palliative care to those in need. During a pandemic, palliative care teams must respond to evolving needs by rapidly reallocating resources, devising symptom management strategies, providing training for non-professionals, facilitating transitions from inpatient to community resources, and effectively utilizing standardized data collection systems to monitoring patient conditions and needs (13, 17). This enables healthcare personnel to establish and sustain demand-responsive palliative care under the pressures of a pandemic, which is crucial. Therefore, this research aims to investigate the differences in palliative care needs and discharge planning services requirements among inpatients in Taiwan before and after the COVID-19 pandemic. It also seeks to explore the factors associated with high palliative care needs and death. The goal is to benefit a larger population of end-of-life inpatients, provide insights for policy formulation, and foster peer sharing and mutual learning for continued growth.

## 2 Methods

### 2.1 Design and participants

This research employed a retrospective case–control design and obtained ethical approval from the Institutional Review Board of Taipei City Hospital (Case Number: TCHIRB-10906013-E). All patient information was accessible only by personnel participating in the study, and all data has been anonymized to protect patient confidentiality. Data were extracted from the Healthcare Information System (HIS) of a regional teaching hospital in Taipei City. The data collection period encompassed two phases: from January 1, 2019, to May 31, 2019, before the COVID-19 pandemic, and from January 1, 2020, to May 31, 2020, during the pandemic.

A total of 231 end-of-life inpatients were collected as the case group, and the control group was created by matching individuals from case group based on age and sex, following a 1:1 ratio with the case group. Inclusion criteria required patients to meet the criteria for high-risk discharge planning services and have a palliative care needs assessment score of ≥4. Data collected included demographic information, palliative care needs assessments and discharge planning services, all of which were analyzed using SPSS 21 software.

### 2.2 The demographic

The demographic variables in this study include: (1) Age, the age of patients at the time of hospitalization; (2) Gender, male or female; (3) Primary Diagnosis, classified using ICD-10 codes; (4) Pre-hospitalization Living Environment, categorized as living with family, residing in a long-term care facility, or living alone; (5) Death, whether the patient died during the hospitalization; (6) Hospitalization Length, the average length of hospital stay.

### 2.3 Palliative care needs assessment

The palliative care needs assessment score in this research was developed based on key indicators related to palliative care discussions at a regional teaching hospital in Taipei City, following the framework established by Weissman and Meier in 2011 (18). Ultimately, a palliative care needs assessment tool customized to the specific hospital’s requirements was developed (Supplementary Figure S1).

The content of the palliative care needs assessment tool includes: (1) Category A: Major conditions related to the current hospitalization and palliative care, including advanced-stage cancer, end-stage recurrent cancer, end-stage chronic obstructive pulmonary disease (COPD), end-stage liver disease, over 2 years of dialysis, end-stage heart disease, severe neurological conditions, life-threatening acute illnesses, etc. Each item is scored with 2 points; (2) Category B: Comorbidities for this hospitalization, encompassing primary cancer, moderate COPD, cirrhosis, moderate heart failure, other complex illnesses, etc. Each item is scored with 1 point, and there should be no overlap in selection with Category A; (3) Category C: Assessment of the patient’s Activities of Daily Living (ADL), scored from 1 to 3 points; (4) Category D: Other conditions, including whether the patient is experiencing intolerable pain, unmanageable psychological issues, repeated emergency department visits within 30 days, or transfers from the intensive care unit. Each item is scored with 1 point.

The palliative care needs assessment score is calculated by summing the scores from the aforementioned A, B, C, and D categories. When the palliative care needs assessment score is ≥4 points, it indicates a need for palliative care (19). In Taiwan, to respect the medical wishes of terminally ill patients and protect their rights, the Hospice Palliative Care Act was enacted, requiring a diagnosis by two physicians to confirm a patient as end of life. Additionally, our hospital has included end of life patients in the high-risk cases for discharge planning services to ensure comprehensive discharge preparation.

### 2.4 Discharge planning

The content of the discharge planning services includes: (1) Assessment of ADL, comprising 10 items. The scores are systematically categorized into independent (≥60 points), partially dependent (40–59 points), and fully dependent (≤39 points); (2) Support system, distinguishing between self-care or requires a caregiver;(3) Skin condition, distinguishing between none, pressure ulcer and wound or Stoma (4) Tube care, distinguishing between no tubes, one tube, and two or more tubes; (5) Resource referrals, categorized as no need, capable of seeking resources independently, or requiring referrals to long-term care-related resources; (6) Discharge arrangements, categorized as returning home, placement in long-term care facilities after discharge, or having no post-discharge arrangements; (7) Nursing guidance: Includes various types of guidance provided to patients, such as ambulation, disease awareness, suction, and others; (8) Assistive equipment: Includes various types of medical equipment such as hospital beds, wheelchairs, walkers, oxygen concentrators, and others. The assessment is conducted by the primary nursing care nurse upon admission, A score of ≥5 points is categorized as “high-risk case,” and a reassessment is conducted every 72 h to confirm individual needs. Additionally, if a patient scores less than 5 points but meets the following criteria: hospitalization length > 14 days, hip joint surgery, stroke, brain injury or spinal cord injury, individuals with an ADL score ≥ 2 points, those with transfer needs, referral resource score ≥ 2 points, discharge planning assessment score ≥ 2 points, or those with indwelling tubes, they are included in the high-risk case management, and continuous follow-up is carried out by the dedicated nurse for discharge planning services.

### 2.5 Data analysis

Data collected in this research will be subjected to statistical analysis using SPSS 21.0. The continuous variables were displayed as mean ± standard deviation (SD), including age, hospitalization length, the palliative care needs assessment score, and the discharge planning assessment score, and categorical variables as case number (n) and percentage (%), including gender, primary diagnosis, pre-hospitalization living environment, death, activities of daily living (ADL), support system, skin condition, tube care, resource referrals, discharge arrangements, nursing guidance, and assistive equipment use. Inferential statistics will involve independent sample t-tests for analyzing continuous variables and chi-square tests for analyzing categorical variables, highlighting differences between the case and control groups. Furthermore, multiple logistic regression analysis will be conducted to identify significant variables associated with high palliative care needs and death, and to calculate the odds ratios as measures of risk factors.

## 3 Results

### 3.1 Comparison between the case and control groups

A total of 462 participants, with 231 individuals in each group. In the case group, there were 151 males (65.4%) and 81 females (34.6%), with an average age of 77.1 ± 13.4 years. In the control group, there were 143 males (62.0%) and 88 females (38.0%), with an average age of 77.3 ± 14.4 years. There was a significant difference in respiratory failure and related symptoms (*p* = 0.004), pre-hospitalization living environment (*p* = 0.017), palliative care needs assessment scores (*p* = 0.010), with the case group averaging 5.36 ± 1.24 points and the control group averaging 5.08 ± 1.08 points (Table 1).

**Table 1:** Demographic information and palliative care needs assessment score.

Variables, n(%)	Case group *n* = 231	Control group *n* = 231	*t*	X^2^	*p*-value
Male	151(65.4%)	143(62.0%)		0.60	0.439
Age (Mean ± SD)	77.1 ± 13.4	77.3 ± 14.4	−0.18		0.854
**Diagnosis**					
Pneumonia or other lung diseases	50 (21.6%)	57 (24.7%)		0.60	0.440
Septicemia	31 (13.4%)	28 (12.1%)		0.18	0.676
Cancer	19 (8.2%)	23 (10.0%)		0.42	0.517
Respiratory failure	30 (13.0%)	12 (5.2%)		8.49	0.004**
Bleeding	11 (4.8%)	17 (7.4%)		1.37	0.242
Infections	17 (7.4%)	20 (8.7%)		0.26	0.607
Kidney failure or other kidney diseases	9 (3.9%)	5 (2.2%)		1.18	0.278
Fever	12 (5.2%)	6 (2.6%)		2.08	0.149
Cerebral infarction or cerebrovascular disease	9 (3.9%)	7 (3.0%)		0.26	0.611
Heart failure or other heart diseases	7 (3.0%)	10 (4.3%)		0.55	0.458
Diabetes or metabolic and endocrine disorders	10 (4.3%)	4 (1.7%)		2.65	0.103
Living environment				8.20	0.017*
With family	116 (50.2%)	144 (62.3%)			
Long-term care facility	96 (41.6%)	67 (29.0%)			
Solitary	19 (8.2%)	20 (8.7%)			
Death	82 (35.5%)	91 (39.4%)		0.75	0.387
Hospitalization length^b^, Mean ± SD	19.5 ± 12.3	20.9 ± 15.03	−1.04		0.295
Readmission	21 (9.1%)	19 (8.2%)		0.11	0.741
Palliative care needs assessment score, Mean ± SD	5.36 ± 1.24	5.08 ± 1.08	2.60		0.010*

SD, standard deviation. t, Independent Samples *t*-test; X^2^, Chi-Square Test of Independence. *p < 0.05; **p < 0.01; ***p < 0.001.

The comparison of discharge planning services of the case and control groups revealed that discharge planning assessment scores, mortality status, length of hospital stay, readmission, ADL, skin condition, tube care, resource referral, and discharge placement all showed no significant differences and were homogenous between the two groups (Table 2).

**Table 2:** Discharge planning assessment.

Variables, n(%)	Case group *n* = 231	Control group *n* = 231	*t*	X^2^	*p*-value
Discharge planning assessment score (Mean ± SD)	4.87 ± 1.97	4.65 ± 1.911	1.17		0.241
Activity of Daily Living (ADL)				0.19	0.908
Fully independent	10 (4.3%)	12 (5.20%)			
Mostly dependent	23 (10.0%)	23 (10.0%)			
Fully dependent	198 (85.7%)	196 (84.8%)			
Have supporting system	195 (84.4%)	208 (90.0%)		3.28	0.145
Skin condition				3.64	0.162
None	159(68.8%)	170 (73.6%)			
pressure ulcer	45 (19.5%)	30 (13.0%)			
Wound or stoma	27 (11.7%)	31(13.4%)			
Have tube care	186 (80.5%)	193 (83.5%)		0.72	0.396
Need resources referral	68 (29.4%)	74 (32.0%)		0.37	0.545
Discharge placement				4.42	0.110
Home	118 (51.1%)	138 (59.7%)			
Long-term care facility	100 (43.3%)	86(37.2%)			
None	13 (5.6%)	7 (3.0%)			

SD, standard deviation. t, Independent Samples t-test; X^2^, Chi-Square Test of Independence. *p < 0.05; **p < 0.01; ***p < 0.001.

Among the nursing instructions and assistive device utilization assessments, statistically significant differences were observed in specific aspects of patient care. These included “Nasogastric tube feeding and care” (*p* = 0.002), “Steam inhalation” (*p* = 0.003), “Turning and positioning” (*p* < 0.001), “Percussion” (*p* < 0.001), “Passive range of motion” (*p* < 0.001), and “Blood pressure measurement” (*p* < 0.001). Regarding the assistive device utilization assessments, there was a statistically significant difference in the utilization of “hospital beds” (*p* < 0.001), while no significant differences were observed in other aspects (Table 3).

**Table 3:** Nursing guidance and assistive equipment.

Variables, n(%)	Case group *n* = 231	Control group *n* = 231	*t*	X^2^
**Nursing guidance**				
Disease awareness	180 (77.9%)	172 (74.5%)	0.76	0.382
Catheter care	48 (20.8%)	35 (15.2%)	2.48	0.115
Tracheostomy care	6 (2.6%)	8 (3.5%)	0.30	0.587
Wound or stoma care	22 (9.5%)	31 (13.4%)	1.73	0.189
Nasogastric tube feeding and care	86 (37.2%)	56 (24.2%)	9.15	0.002**
Steam inhalation	24 (10.4%)	8 (3.5%)	8.60	0.003**
Suction	35 (15.2%)	28 (12.1%)	0.90	0.343
Turning and positioning	113 (48.9%)	65 (28.1%)	21.06	<0.001***
Percussion	93 (40.3%)	37 (16.0%)	33.57	<0.001***
Passive range of motion	93 (40.3%)	49 (21.2%)	19.68	<0.001***
Early ambulation	24 (10.4%)	35 (15.2%)	2.35	0.125
Breathing training	5 (2.2%)	1 (0.4%)	2.70	0.100
Glucose measurement	21 (9.1%)	13 (5.6%)	2.03	0.154
Blood pressure measurement	126 (54.5%)	58 (25.1%)	41.76	<0.001***
**Assistive equipment**				
Bed	118 (51.1%)	34 (14.7%)	69.18	<0.001***
Medical air mattress	6 (2.6%)	8 (3.5%)	0.30	0.587
Wheelchair	56 (24.2%)	39 (16.9%)	3.83	0.050
Walker	2 (0.9%)	3 (1.3%)	0.20	0.653
Oxygen concentrator	8 (3.5%)	8 (3.5%)	0	1.000
Suction machine	11 (4.8%)	9 (3.9%)	0.21	0.648
Steam inhaler	7 (3.0%)	3 (1.3%)	1.64	0.201
Glucose monitor	4 (1.7%)	3 (1.3%)	0.15	0.703
Sphygmomanometer	11 (4.8%)	5 (2.2%)	2.33	0.127

X^2^, Chi-Square Test of Independence. *p < 0.05; **p < 0.01; ***p < 0.001.

### 3.2 Physician assessment of end-of-life case

In the section of palliative care needs assessment, in the case group, a total of 114 cases confirmed by physicians as end-of-life cases. Among them, 30 individuals (26.3%) had a palliative care needs assessment score of 4 points, and 84 individuals (73.7%) scored 5 or higher. In the control group, a total of 101 cases were confirmed by physicians as end-of-life cases. Among them, 31 individuals (40.6%) had a palliative care needs assessment score of 4 points, and 60 individuals (59.4%) scored 5 or higher. A chi-square test was used to analyze whether there was a difference in the proportion of patients assessed by physicians as end-of-life cases between the case group and the control group. The results indicated a significant difference (*p* = 0.026). This means that during the pandemic, there was a significantly higher proportion of inpatients with palliative care needs assessment scores of 5 or higher who were assessed by physicians as end-of-life cases compared to those assessed before the pandemic (Table 4).

**Table 4:** Comparison of palliative care needs assessment score of all end-of-life cases assessed by physician.

Variables	Case group *n* = 114 n(%)	Control group *n* = 101 n(%)	X^2^	*p*
palliative care needs assessment score			4.936	0.026*
4	30 (26.3%)	41 (40.6%)		
>4	84 (73.7%)	60 (59.4%)		
death status			1.238	0.266
death	29 (25.4%)	22 (19.3%)		
alive	85 (74.6%)	92 (80.7%)		

X^2^, Chi-Square Test of Independence. *p < 0.05; **p < 0.01; ***p < 0.001.

Regarding the death status, in the case group, out of the 114 patients assessed by physicians as end-of-life cases (49.35%), there were 29 deaths (25.4%). In the control group, out of 101 patients assessed by physicians as end-of-life cases (43.72%), there were 22 deaths (19.3%). A chi-square test was used to analyze whether there was a difference in the proportion of patients assessed by physicians as end-of-life cases in the case group and control group. The results indicated no significant difference (*p* = 0.266; Table 4).

### 3.3 Logistic regression analysis

In the case group, there were 82 patients with palliative care needs assessment scores of 4 points, and the number of deaths was 21 (25.6%). There were 149 patients with palliative care needs assessment scores ≥5 points, the number of deaths was 61 (40.9%). A chi-squared test was used to analyze whether there was a difference in the proportion of patients with palliative care needs assessment scores of 4 points and ≥ 5 points in the case group. The results indicated that the mortality rate was significantly higher for individuals with a palliative care needs assessment score of ≥5 points compared to those with a score of 4 points (*p* = 0.020); In the control group, there were 126 patients with palliative care needs assessment scores of 4 points, and the number of deaths was 42 (33.3%). There were 105 patients with palliative care needs assessment scores ≥5 points, the number of deaths was 49 (46.7%). A chi-squared test was used to analyze whether there was a difference in the proportion of patients with palliative care needs assessment scores of 4 points and ≥ 5 points in the control group. The results indicated that the mortality rate was significantly higher for individuals with a palliative care needs assessment score of ≥5 points compared to those with a score of 4 points (*p* = 0.039).

Therefore, a palliative care needs assessment score of 4 points was categorized as the low palliative care needs group, while a score of ≥5 points was considered the high palliative care needs group. Subsequently, the factors associated to high palliative care needs and deaths were analyzed separately for the case group and the control group, as outlined below.

#### 3.3.1 Case group

**Figure 1:**
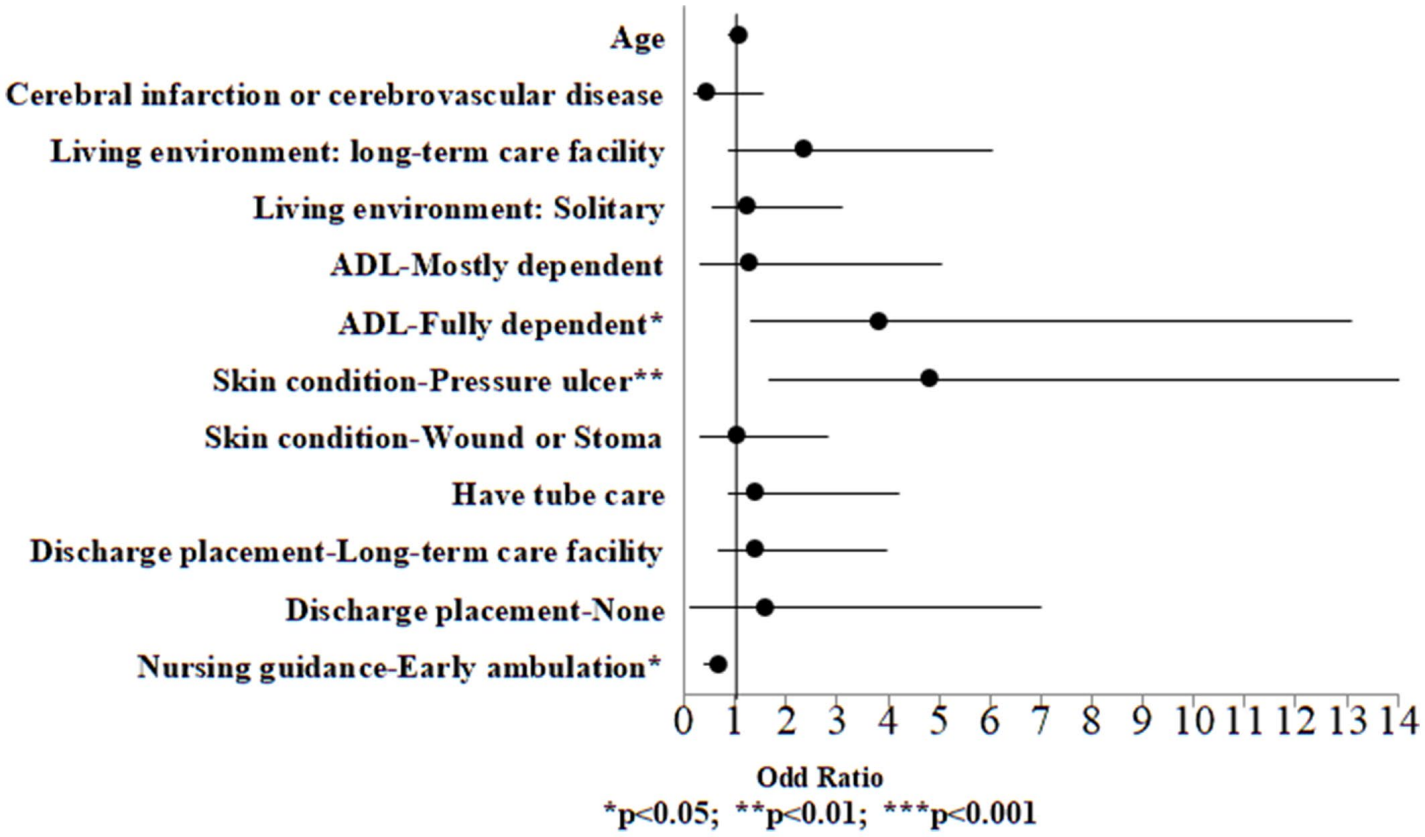
Odds ratio plot of high palliative care needs (case group).

Among the case group, univariate logistic regression analysis was performed on all variables, including demographic information and discharge planning services to examine the variables associated to high palliative care needs. Significant variables from the univariate logistic regression analysis (*p* < 0.05) were included in the multivariate logistic regression model to identify the risk of high palliative care needs. The results indicated that in the case group, individuals who were fully dependent in ADL exhibited a 3.77-fold higher risk compared to those who were fully independent (OR: 3.77, 95% CI: 1.31, 13.11, *p* = 0.018). Moreover, individuals with pressure ulcers exhibited a 4.76-fold increased risk relative to those without any wounds (OR: 4.76, 95% CI: 1.67, 21.12, *p* = 0.003). Conversely, those who received ambulation guidance exhibited a 0.65-fold lower risk compared to those who did not receive such guidance (OR: 0.65, 95% CI: 0.39, 0.81, *p* = 0.032; Table 5; Figure 1).

**Table 5:** Univariate and multivariate logistic regression analyses of high palliative care needs (case group).

Variables	Univariate analysis		Multivariate analysis	
	Odds ratio	*p* value	Odds ratio	*p* value
Male	0.91(0.40, 2.80)	0.907		
Age	1.03(1.02, 1.05)	0.009**	1.02 (0.87, 1.08)	0.582
**Diagnosis**				
Pneumonia or other lung diseases	1.20 (0.77, 2.36)	0.400		
Septicemia	1.27 (0.68, 4.67)	0.309		
Cancer	0.68 (0.33, 1.23)	0.181		
Respiratory failure	1.55 (0.88, 3.77)	0.392		
Bleeding	0.31 (0.23, 1.11)	0.090		
Infections	2.10 (0.88, 3.67)	0.122		
Kidney failure or other kidney diseases	0.55 (0.25, 2.31)	0.445		
Fever	2.83 (0.64, 12.49)	0.17		
Cerebral infarction or cerebrovascular diseases	0.29 (0.11, 0.69)	0.007**	0.41 (0.19, 1.55)	0.158
Heart failure or other heart diseases	1.33 (0.51, 3.50)	0.751		
Diabetes or metabolic and endocrine disorders	3.98 (0.66, 12.71)	0.459		
**Living environment**				
With family	1		1	
Long-term care facility	3.83(2.28, 6.95)	<0.001***	2.31 (0.88, 6.06)	0.121
Solitary	0.28 (0.06, 2.57)	0.212	1.21 (0.54, 3.12)	0.811
Hospitalization length	1.01 (0.98, 1.04)	0.549		
Readmission	0.58 (0.28, 1.28)	0.231		
Discharge planning assessment score	1.06 (0.87, 1.29)	0.553		
**Activity of Daily Living (ADL)**				
Fully independent	1		1	
Mostly dependent	1.53 (0.55, 4.99)	0.289	1.22 (0.31, 5.07)	0.632
Fully dependent	7.54 (3.37, 20.66)	<0.001***	3.77 (1.31, 13.11)	0.018*
Have a support system	0.62(0.17, 2.18)	0.45		
**Skin condition**				
None	1		1	
Pressure ulcer	3.89 (1.57, 8.99)	0.002**	4.76 (1.67, 21.12)	0.003**
Wound or Stoma	1.12 (0.81, 2.89)	0.284	1.01 (0.31, 2.81)	0.837
Have tube care	2.33 (1.99, 5.12)	<0.001***	1.35 (0.89, 4.21)	0.187
Need resources referral	1.01 (0.64, 1.60)	0.951		
**Discharge placement**				
Home	1		1	
Long-term care facility	3.12 (2.21, 5.78)	<0.001***	1.35 (0.66, 3.99)	0.403
None	0.91 (0.20, 2.11)	0.753	1.55 (0.11, 7.01)	0.831
**Nursing guidance**				
Disease awareness	078 (0.21, 1.11)	0.101		
Catheter care	1.11 (0.77, 2.88)	0.666		
Tracheostomy care	0.32 (0.15, 1.31)	0.155		
Wound or stoma care	0.51 (0.31, 1.20)	0.581		
Nasogastric tube feeding and care	1.55 (0.98, 3.02)	0.177		
Steam inhalation	1.21 (0.52, 2.95)	0.612		
Suction	1.88 (0.88, 4.21)	0.052		
Turning and positioning	1.55 (0.87, 2.35)	0.115		
Percussion	1.88 (0.85, 2.77)	0.352		
Passive range of motion	1.31 (0.77, 1.99)	0.224		
Ambulation	0.87 (0.44, 0.98)	0.011*	0.65 (0.39, 0.81)	0.032*
Breathing training	1.21 (0.22, 11.84)	0.62		
Glucose measurement	0.88 (0.33, 3.10)	0.997		
Blood pressure measurement	1.22 (0.82, 2.12)	0.664		

OR, odds ratio. *p < 0.05; **p < 0.01; ***p < 0.001.

**Figure 2:**
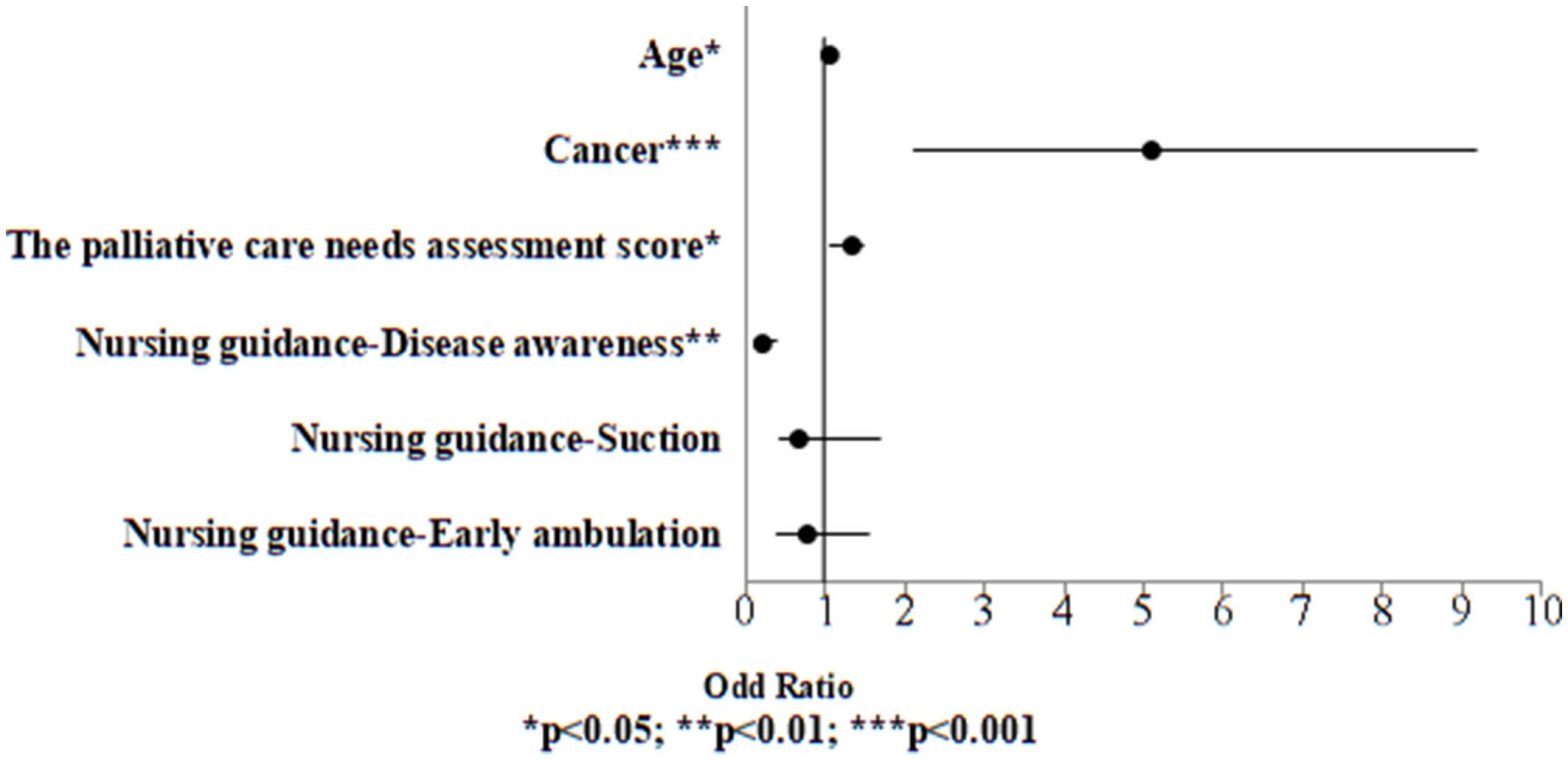
Odds ratio plot of death (case group).

Univariate logistic regression analysis was performed on all variables, including demographic information, palliative care needs assessment score and discharge planning services to examine the variables associated to death. Significant variables from the univariate logistic regression analysis (*p* < 0.05) were included in the multivariate logistic regression model to identify the risk of death. The results indicated that in the case group, the risk exhibited a 1.06-fold higher risk for 1 year older in age (OR: 1.06, 95% CI: 1.01, 1.09, *p* = 0.021). Individuals with cancer exhibited a 5.12-fold increased risk relative to those who were without cancer (OR: 5.12, 95% CI: 2.11, 9.18, *p* < 0.001). The risk exhibited a 1.35-fold higher risk for one point higher in palliative care needs assessment score (OR: 1.35, 95% CI: 1.05, 1.49, *p* = 0.011). Conversely, those who received disease awareness guidance exhibited a 0.22-fold lower risk compared to those who did not receive such guidance (OR: 0.22, 95% CI: *p* < 0.001) (Table 6; Figure 2).

**Table 6:** Univariate and multivariate logistic regression analyses of death (case group).

Variables	Univariate analysis		Multivariate analysis	
	Odds ratio	*p* value	Odds ratio	*p* value
Male	1.33 (0.88, 1.95)	0.318		
Age	1.07 (1.02, 1.09)	0.015*	1.06 (1.01, 1.09)	0.021*
**Diagnosis**				
Pneumonia or other lung diseases	1.21 (0.81, 2.12)	0.870		
Septicemia	1.12 (0.63, 3.11)	0.573		
Cancer	5.18 (3.34, 9.28)	<0.001***	5.12 (2.11, 9.18)	<0.001***
Respiratory failure	1.09 (0.65, 2.81)	0.589		
Bleeding	0.45 (0.31, 1.21)	0.190		
Infections	0.47 (0.11, 2.02)	0.311		
Kidney failure or other kidney diseases	1.62 (0.63, 4.12)	0.700		
Fever	1.08 (0.41, 2.88)	0.901		
Cerebral infarction or cerebrovascular diseases	0.66 (0.16, 1.88)	0.402		
Heart failure or other heart diseases	0.72 (0.32, 1.89)	0.355		
Diabetes or metabolic and endocrine disorders	0.66 (0.21, 2.24)	0.509		
**Living environment**				
With family	1			
Long-term care facility	1.02 (0.57, 1.51)	0.998		
Solitary	0.45 (0.21, 1.38)	0.264		
Palliative care needs assessment score	1.33 (1.12, 1.55)	0.008**	1.35 (1.05, 1.49)	0.011*
Hospitalization length	0.89 (0.88, 1.01)	0.225		
Readmission	1.00 (0.51, 1.96)	0.994		
Discharge planning assessment score	1.16 (0.86, 1.37)	0.245		
**Activity of Daily Living (ADL)**				
Fully independent	1			
Mostly dependent	2.88 (0.79, 7.95)	0.167		
Fully dependent	3.12 (0.91, 7.58)	0.087		
Have supporting system	1.17 (0.67, 2.04)	0.997		
Skin condition				
None	1			
Pressure ulcer	1.85 (0.95, 2.55)	0.061		
Wound or Stoma	0.79 (0.38, 1.28)	0.759		
Have tube care	0.88 (0.48, 1.56)	0.888		
Need resources referral	1.33 (0.88, 1.95)	0.321		
**Discharge placement**				
Home	1			
Long-term care facility	1.21 (0.67, 1.47)	0.988		
None	0.66 (0.33, 1.87)	0.514		
**Nursing guidance**				
Disease awareness	0.18(0.17, 0.28)	<0.001***	0.22 (0.16, 0.38)	<0.001***
Catheter care	0.58 (0.41, 1.32)	0.205		
Tracheostomy care	1.00 (0.35, 3.31)	0.902		
Wound or stoma care	0.66 (0.36, 1.01)	0.052		
Nasogastric tube feeding and care	0.80(0.46, 1.07)	0.099		
Steam inhalation	0.77 (0.32, 1.81)	0.454		
Suction	0.68 (0.36, 0.98)	0.021*	0.66 (0.41, 1.68)	0.188
Turning and positioning	0.71 (0.46, 1.11)	0.067		
Percussion	0.75 (0.47, 1.13)	0.155		
Passive range of motion	0.77 (0.50, 1.12)	0.198		
Ambulation	0.45 (0.25, 0.98)	0.021*	0.78 (0.40, 1.55)	0.235
Breathing training	0.83 (0.15, 4.56)	0.834		
Glucose measurement	0.75 (0.38, 1.55)	0.665		
Blood pressure measurement	0.69 (0.40, 1.01)	0.055		

OR, odds ratio. *p < 0.05; **p < 0.01; ***p < 0.001.

#### 3.3.2 Control group

**Figure 3:**
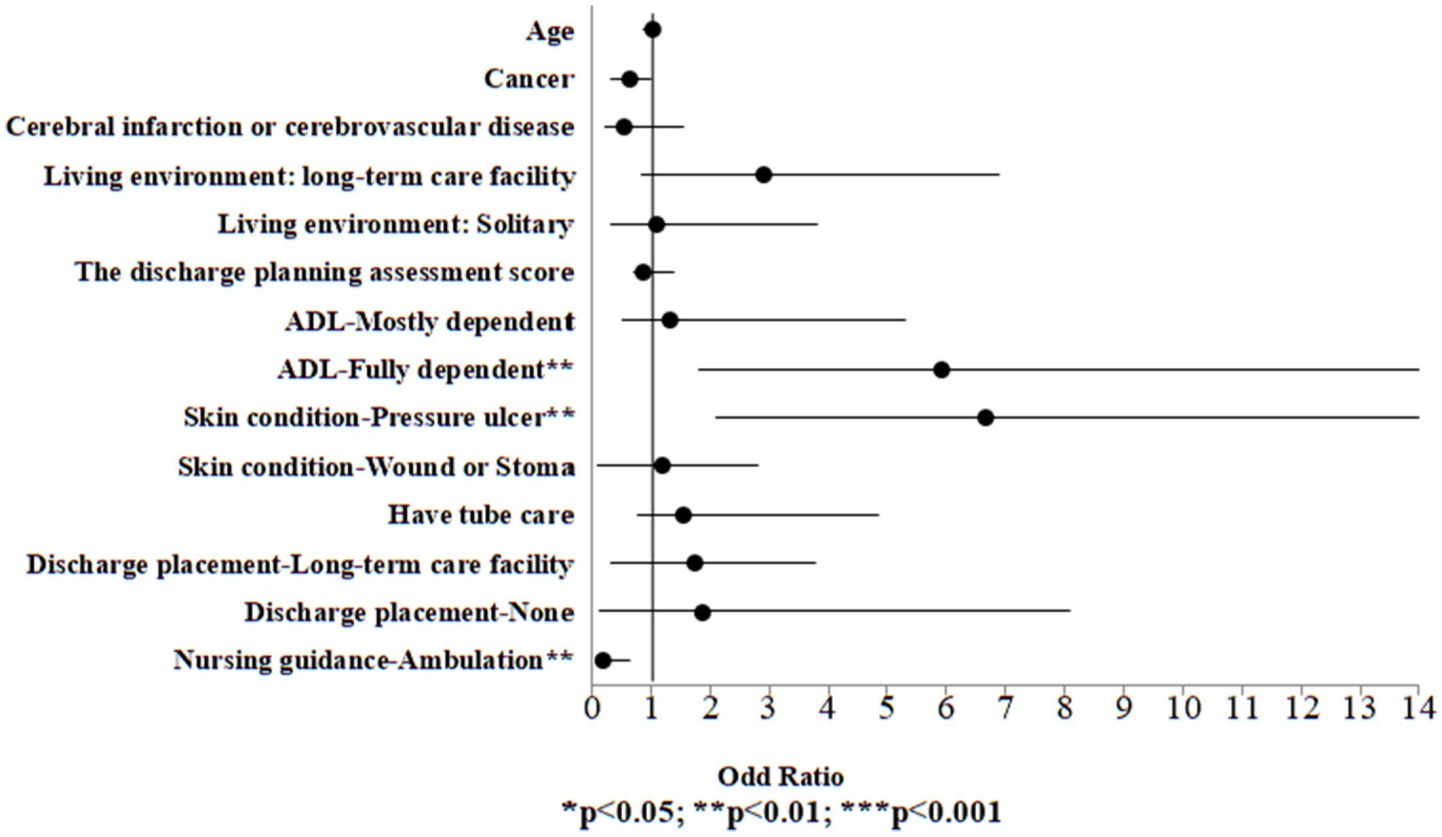
Odds ratio plot of high palliative care needs (control group).

During COVID-19 pandemic, among the control group, univariate logistic regression analysis was performed on all variables, including demographic information and discharge planning services to examine the variables associated to high palliative care needs. Significant variables from the univariate logistic regression analysis (*p* < 0.05) were included in the multivariate logistic regression model to identify the risk of high palliative care needs. The results indicated that in the case group, individuals who were fully dependent in ADL exhibited a 5.91-fold higher risk compared to those who were fully independent (OR:5.91, 95% CI:1.81, 14.13, *p* = 0.008). Individuals with pressure ulcers exhibited a 6.66-fold increased risk relative to those without any wounds (OR:6.66, 95% CI:2.10, 15.22, *p* = 0.001). Conversely, those who received ambulation guidance exhibited a 0.19-fold lower risk compared to those who did not receive such guidance (OR:0.19, 95% CI:0.10, 0.65, *p* = 0.002; Table 7; Figure 3).

**Table 7:** Univariate and multivariate logistic regression analyses of high palliative care needs (control group).

Variables	Univariate analysis		Multivariate analysis	
	Odds ratio	*p* value	Odds ratio	*p* value
Male	0.66(0.50, 1.21)	0.211		
Age	1.02(1.01, 1.04)	0.007**	1.02 (0.87, 1.05)	0.377
**Diagnosis**				
Pneumonia or other lung diseases	1.11 (0.77, 2.36)	0.412		
Septicemia	1.32 (0.68, 4.67)	0.382		
Cancer	3.55 (1.10, 6.98)	0.009**	0.66 (0.31, 1.01)	0.051
Respiratory failure	1.92 (0.88, 3.77)	0.351		
Bleeding	0.52 (0.23, 1.11)	0.099		
Infections	3.11 (0.88, 3.67)	0.211		
Kidney failure or other kidney diseases	0.65 (0.25, 2.31)	0.214		
Fever	2.11 (0.34, 13.54)	0.098		
Cerebral infarction or cerebrovascular diseases	0.21 (0.05, 0.71)	0.009**	0.55 (0.21, 1.55)	0.231
Heart failure or other heart diseases	1.52 (0.61, 3.77)	0.791		
Diabetes or metabolic and endocrine disorders	4.12 (0.66, 12.99)	0.411		
**Living environment**				
With family	1		1	
Long-term care facility	4.89.(2.58, 8.25)	<0.001***	2.91 (0.83, 6.88)	0.121
Solitary	0.33 (0.05, 3.11)	0.328	1.11 (0.31, 3.82)	0.788
Hospitalization length	1.02 (0.97, 1.04)	0.209		
Readmission	0.78 (0.33, 1.31)	0.156		
Discharge planning assessment score	1.55 (1.22, 1.89)	<0.001***	0.88 (0.70, 1.39)	0.665
**Activity of Daily Living (ADL)**				
Fully independent	1		1	
Mostly dependent	2.01 (0.65, 4.89)	0.312	1.32 (0.51, 5.32)	0.532
Fully dependent	9.88 (4.37, 25.55)	<0.001***	5.91 (1.81, 14.13)	0.008**
Have a support system	0.62(0.17, 2.18)	0.45		
**Skin condition**				
None	1		1	
Pressure ulcer	5.55 (2.11, 9.82)	0.001**	6.66 (2.10, 15.0.22)	0.001**
Wound or Stoma	0.52 (0.24, 2.99)	0.524	1.21 (0.11, 2.80)	0.878
Have tube care	2.98 (1.12, 3.91)	<0.001***	1.55 (0.79, 4.87)	0.842
Need resources referral	1.01 (0.64, 1.60)	0.951		
**Discharge placement**				
Home	1		1	
Long-term care facility	3.99 (2.15, 6.44)	<0.001***	1.75 (0.32, 3.80)	0.553
None	0.88 (0.19, 3.12)	0.760	1.88 (0.12, 8.08)	0.811
**Nursing guidance**				
Disease awareness	0.78 (0.21, 1.11)	0.101		
Catheter care	1.11 (0.77, 2.88)	0.666		
Tracheostomy care	0.33 (0.15, 1.31)	0.155		
Wound or stoma care	0.2 (0.31, 1.20)	0.581		
Nasogastric tube feeding and care	1.555 (0.98, 3.02)	0.120		
Steam inhalation	1.42 (0.88, 2.27)	0.699		
Suction	1.24 (0.52, 2.95)	0.077		
Turning and positioning	1.97 (0.97, 4.01)	0.099		
Percussion	1.45 (0.93, 2.25)	0.121		
Passive range of motion	1.82 (0.77, 1.99)	0.334		
Early ambulation	0.12 (0.08, 0.99)	0.001**	0.19 (0.10, 0.65)	0.002**
Breathing training	1.21 (0.22, 11.84)	0.120		
Glucose measurement	0.88 (0.33, 3.10)	0.725		
Blood pressure measurement	1.02 (0.88, 1.42)	0.659		

OR, odds ratio. *p < 0.05; **p < 0.01; ***p < 0.001.

**Figure 4:**
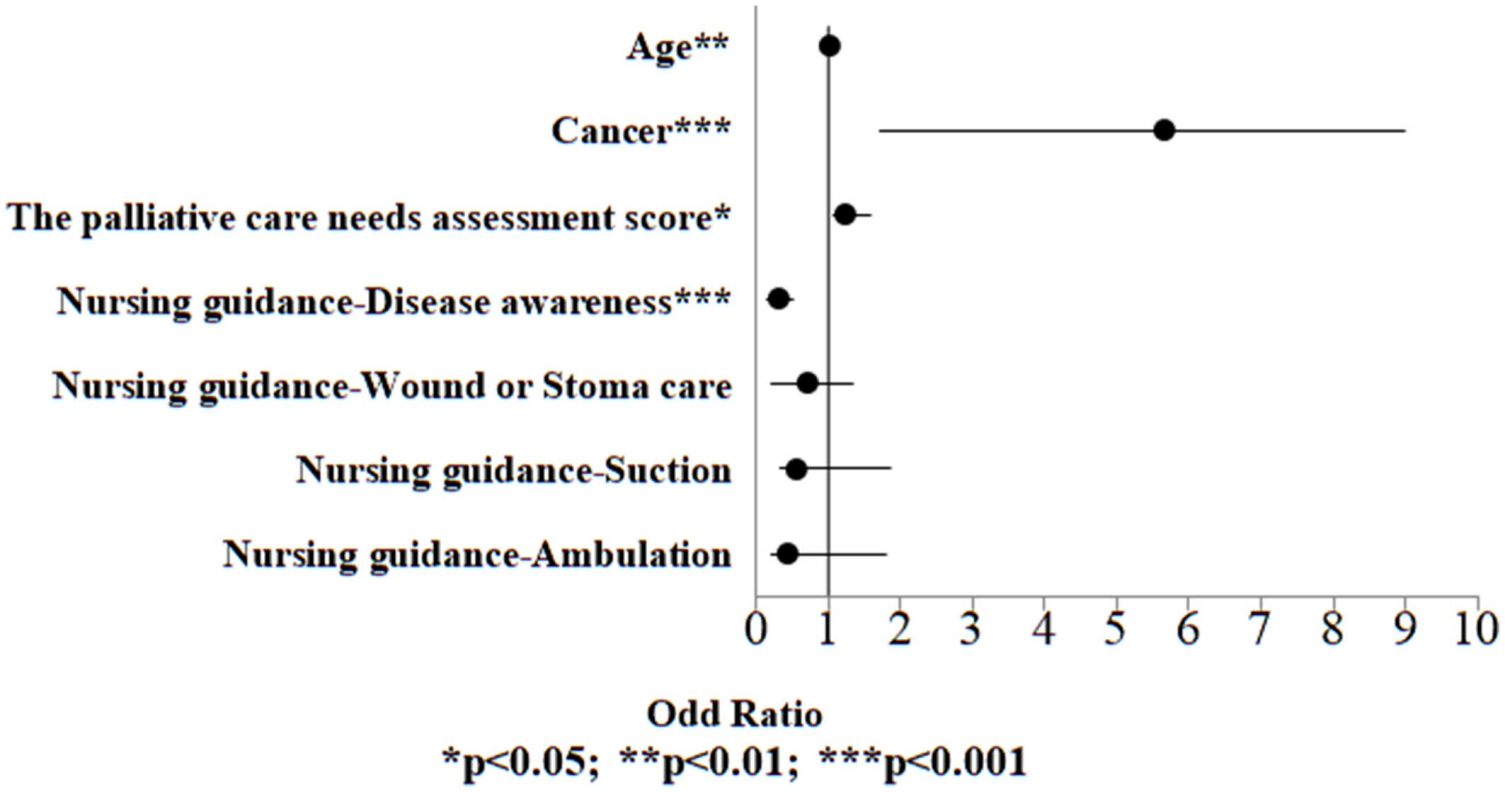
Odds ratio plot of death (control group).

Univariate logistic regression analysis was performed on all variables, including demographic information, palliative care needs assessment score and discharge planning services to examine the variables associated to death. Significant variables from the univariate logistic regression analysis (*p* < 0.05) were included in the multivariate logistic regression model to identify the risk of death. The results indicated that in the case group, the risk exhibited a 1.01-fold higher risk for 1 year older in age (OR:1.01, 95% CI:1.00, 1.05, *p* = 0.009). Individuals with cancer exhibited a 5.66-fold increased risk relative to those who were without cancer (OR:5.66, 95% CI:1.70, 9.01, *p* < 0.001). The risk exhibited a 1.21-fold higher risk for one point higher in palliative care needs assessment score (OR:1.21, 95% CI:1.06, 1.58, *p* = 0.013). Conversely, those who received disease awareness guidance exhibited a 0.29-fold lower risk compared to those who did not receive such guidance (OR:0.29, 95% CI:0.15, 0.51, *p* < 0.001; Table 8; Figure 4).

**Table 8:** Univariate and multivariate logistic regression analyses of death (control group).

Variables	Univariate analysis		Multivariate analysis	
	Odds ratio	*p* value	Odds ratio	*p* value
Male	1.11 (0.70, 1.75)	0.433		
Age	1.01 (1.00, 1.04)	0.008**	1.01 (1.00, 1.05)	0.009**
**Diagnosis**				
Pneumonia or other lung diseases	1.01 (0.66, 1.72)	0.867		
Septicemia	1.33 (0.75, 3.13)	0.599		
Cancer	5.86 (3.31, 9.84)	<0.001***	5.66 (1.70, 9.01)	<0.001***
Respiratory failure	0.55 (0.17, 1.72)	0.302		
Bleeding	0.50 (0.16, 1.57)	0.235		
Infections	0.66 (0.20, 2.14)	0.489		
Kidney failure or other kidney diseases	0.47 (0.11, 2.02)	0.311		
Fever	1.26 (0.43, 3.70)	0.672		
Cerebral infarction or cerebrovascular diseases	1.07 (0.41, 2.80)	0.897		
Heart failure or other heart diseases	0.44 (0.02, 1.35)	0.412		
Diabetes or metabolic and endocrine disorders	0.65 (0.19, 2.35)	0.511		
**Living environment**				
With family	1			
Long-term care facility	1.00(0.77, 1.50)	0.999		
Solitary	0.66 (0.28, 1.32)	0.352		
Palliative care needs assessment score	1.18 (1.01, 1.39)	0.011*	1.21 (1.06, 1.58)	0.013*
Hospitalization length	0.94 (0.58, 1.54)	0.818		
Readmission	1.06 (0.96, 1.17)	0.225		
Discharge planning assessment score	1.00 (0.51, 1.96)	0.994		
**Activity of Daily Living (ADL)**				
Fully independent	1			
Mostly dependent	2.30 (0.55, 9.56)	0.251		
Fully dependent	2.11 (0.55, 9.21)	0.061		
Have supporting system	1.23 (0.82, 1.85)	0.315		
**Skin condition**				
None	1			
Pressure ulcer	1.15 (0.60, 2.20)	0.671		
Wound or Stoma	0.51 (0.24, 1.11)	0.089		
Have tube care	0.47 (0.11, 2.02)	0.311		
Need resources referral	1.26 (0.43, 3.70)	0.672		
**Discharge placement**				
Home	1			
Long-term care facility	1.07 (0.41, 2.80)	0.897		
None	0.55 (0.17, 1.72)	0.302		
**Nursing guidance**				
Disease awareness	0.30(0.19, 0.35)	<0.001***	0.29 (0.15, 0.51)	<0.001***
Catheter care	0.77 (0.41, 1.44)	0.411		
Tracheostomy care	1.42 (0.88, 2.27)	0.148		
Wound or stoma care	0.60 (0.28, 088)	0.039*	0.70 (0.21, 1.35)	0.33
Nasogastric tube feeding and care	0.99 (0.98, 1.01)	0.327		
Steam inhalation	0.73 (0.35, 1.89)	0.455		
Suction	0.38 (0.16, 0.78)	0.023*	0.55 (0.32, 1.88)	0.221
Turning and positioning	0.71 (0.46, 1.11)	0.067		
Percussion	0.50 (0.16, 1.57)	0.235		
Passive range of motion	0.66 (0.20, 2.14)	0.489		
Early ambulation	0.51 (0.21, 0.88)	0.011*	0.44 (0.21, 1.88)	0.181
Breathing training	0.76 (0.50, 1.15)	0.199		
Glucose measurement	0.72 (0.43, 1.20)	0.205		
Blood pressure measurement	0.93 (0.31, 2.81)	0.892		

OR, Odds ratio. *p < 0.05; **p < 0.01; ***p < 0.001.

## 4 Discussion

### 4.1 Comparison before and after the COVID-19 outbreak

The aim of this research is to investigate the palliative care needs and discharge planning services in Taiwan for end-of-life inpatients before and after the COVID-19 pandemic. The results indicated that during the pandemic, a significantly higher proportion of inpatients had a primary diagnosis of “respiratory failure and related symptoms.” Severe COVID-19 patients may develop respiratory failure or ARDS and require respiratory assistance (3, 4, 20, 21).

During the pandemic, there was a significant increase in the percentage of end-of-life inpatients originating from long-term care facilities compared to the period before the pandemic. Long-term care facilities provide specialized, intensive care for individuals with complex medical conditions who require ongoing care. These facilities presented a heightened risk of COVID-19 transmission due to the close proximity of their residents and their medical needs (22, 23). Moreover, patients recovering from COVID-19 often require increased care upon discharge (24, 25). When family members struggle to manage these heightened care needs, they frequently turn to long-term care facilities as their preferred option.

In the discharge planning services, there was a significant increase in the percentage of individuals receive nursing guidance in “nasogastric tube feeding and care,” “steam inhalation,” “suction,” “turning and positioning,” “percussion,” “passive range of motion,” and “blood pressure measurement.” Given that severe COVID-19 patients frequently manifest ARDS and other organ dysfunctions, it is imperative to implement specialized care protocols. These protocols encompass procedures such as intubation and extubation, steam therapy, endotracheal suctioning, positioning, and limb exercises (20, 26).

In nursing rehabilitation for COVID-19 patients, percussion plays a pivotal role to help clear mucus from the lungs and facilitate airway clearance, aiding patients in phlegm removal (27). Most COVID-19 patients admitted to the intensive care unit are at high risk of malnutrition. This risk is primarily associated with severe respiratory infections that trigger inflammation and increased catabolism, leading to elevated energy and protein requirements. Moreover, reduced food intake is frequently observed due to infection-induced anorexia, respiratory distress, indigestion, stress, isolation, and other factors. Therefore, tube feeding is a viable strategy to prevent the worsening of malnutrition (28). Cardiovascular diseases, hypertension, hyperglycemia, and diabetes are all risk factors for COVID-19 infection. These conditions not only worsen the prognosis but also increase the likelihood of severe illness and mortality. Therefore, effective management of blood sugar and blood pressure contributes to reducing the severity of the condition and the mortality (28, 29).

This research revealed that end-of-life inpatients during the pandemic exhibited clinical characteristics of COVID-19 infection or severe COVID-19. To enhance the prognosis and overall health outcomes, it is imperative to focus on the early identification and prediction of COVID-19 disease progression, necessitating increased attention to the care of high-risk cases (30). Patients with COVID-19 may experience a range of virus-related sequelae that necessitate extended stays in the intensive care unit and require respiratory support. To enhance the quality of care and patients’ daily life activities during their hospitalization, it is essential to provide personalized assessments and progressively tailored treatment plans to address their specific medical conditions (21, 31).

### 4.2 Confirmed as end-of-life by physician

Among all inpatients assessed by physicians as end-of-life cases, the case group exhibited a significantly higher proportion of inpatients with palliative care needs assessment scores of 5 points compared to control group. However, there was no significant difference in the inpatient mortality rate between the case group and the control group. This finding may be attributed to the impact of the pandemic, as physicians might exhibit heightened sensitivity toward patients with more severe symptoms, resulting in a more proactive approach to palliative care intervention. However, it might also overlook patients with milder symptoms who still require palliative care intervention.

During the pandemic, there was a shortage of healthcare resources and personnel (14, 17), which exacerbated the challenges associated with delivering palliative care (6, 32). Concurrently, medical resources such as intensive care unit beds, ventilators, and personal protective equipment were in high demand, and healthcare professionals faced the risks of infection and isolation (33, 34). In Taiwan, the comprehensive deployment of nursing personnel for pandemic-related duties has led to the closure or transformation of palliative care units into specialized wards. Consequently, the limited palliative care resources are primarily directed toward patients with severe symptoms, leaving those with milder symptoms with unmet care needs (35). This situation may lead to delays in symptom management, emotional support, and communication for patients with serious illnesses. To ensure continuous palliative care, it is recommended to integrate interdisciplinary professionals and provide enhanced professional training to establish a robust care system that can effectively address the challenges posed by the pandemic (5, 16, 32). This approach is crucial for better addressing the palliative care challenges during pandemics, ensuring that patients receive the appropriate and respectful end-of-life care they need.

In response to the increasing needs for palliative care among inpatients during the pandemic, it is recommended to establish specialized mechanisms and measures for palliative care distinct from acute care. This approach ensures that patients with milder symptoms and those in early disease stages receive the appropriate level of care. Additionally, collaboration with other units to develop management guidelines for COVID-19 symptoms and palliative care consultations is advised, ensuring the provision of care to patients with milder symptoms in the early stages of their illness (31, 36). Furthermore, the lack of recognition and understanding of the value of palliative care has long been a challenge in promoting palliative care (37). To enhance public awareness of palliative care, it is recommended to initiate ACP early to ascertain patients’ preferences for future treatment (7, 38). Promoting ACP in the community allows healthcare professionals ample time for discussions with patients and their families (34). Public media campaigns within the community can effectively stimulate community members’ willingness to participate in ACP (39). Additionally, a range of ACP tools can be made available, including telephone and video consultations (39), smartphone applications and online platforms (40, 41). Furthermore, offering free ACP consultations for vulnerable populations is recommended to enhance public engagement. On the other hand, healthcare professionals often face challenges in initiating ACP discussions and determining the appropriate timing (42). Therefore, it is recommended to provide healthcare professionals with relevant knowledge and training courses on ACP.

### 4.3 Factors associated with high palliative care needs

Both the case and control groups exhibited an increased risk of palliative care needs among inpatients who were assessed as fully dependent in ADL, had pressure ulcers, and did not receive ambulation guidance.

ADL is a measure of maintaining health and home-based routine tasks and activities, encompassing aspects such as eating, drinking, getting in and out of bed, turning over, sitting down and rising, climbing stairs, personal hygiene, dressing, and undressing (43). Palliative care needs assessment inherently includes an evaluation of ADL. Therefore, when a patient is assessed as fully dependent in ADL, their self-care abilities are also compromised, and their palliative care needs are correspondingly higher.

Prolonged bed rest is a primary risk factor for pressure ulcers in inpatients. Failing to reposition patients in a timely manner can lead to discomfort, extended hospital stays, increased healthcare costs, heightened infection risks, and, consequently, life-threatening situations (25). Particularly, providing postoperative ambulation training for patients can enhance physical functionality, reduce postoperative pain, decrease the use of analgesics, shorten hospitalization, and increase the likelihood of a healthy return home (44).

Comparing the case group and the control group, it is evident that although both groups exhibit similar factors associated with high palliative care needs, the risk of high palliative care needs inpatients before the pandemic was higher. This may be attributed to the fact that since the outbreak of COVID-19 in Taiwan, numerous Non-Pharmaceutical Interventions (NPIs) have been developed, resulting in a reduction in medical visits by the public (45). These interventions aimed at preventing the spread of the pandemic and simultaneously controlling the incidence and severity of other notifiable infectious diseases (46). As a result, inpatients during the pandemic generally presented with milder overall symptoms, leading to an overall reduced risk of high palliative care needs.

### 4.4 Factors associated with death

Both the case and control groups exhibited an increased risk of death in inpatients were age, high palliative care needs assessment scores, a diagnosis of cancer, and not receiving disease awareness guidance. Aging is a primary risk factor for many diseases, including cancer, diabetes, cardiovascular diseases, and neurodegenerative disorders. With increasing age, there is a progressive decline in inherent physiological functions, leading to an increased mortality rate (47). Palliative care needs assessment encompasses end-stage diseases, including end-stage cancer, end-stage chronic obstructive pulmonary disease, end-stage heart disease, and other potentially life-limiting conditions (19). Therefore, as the palliative care needs assessment score increases, leading to an increased mortality rate.

In 2019, there were an estimated 23.6 million new cancer cases and 10 million cancer-related deaths worldwide (48). Cancer has consistently ranked as one of the top 10 leading causes of death in Taiwan for many years. Patient education is a practice where healthcare professionals employ techniques such as teaching, counseling, and behavior change to provide systematic learning for patients and their families. It can increase patients’ knowledge, change their attitudes toward the disease, promote healthier behaviors, enhance disease awareness, and improve their understanding of prognosis and related expected quality of life. Patients who are well-informed are more likely to actively participate in their care, resulting in improved treatment outcomes (7).

Comparing the case group and the control group, it was observed that there is no significant difference in the death risk associated with various factors. However, it is essential to note that since the outbreak of COVID-19, various mandatory public health measures have led to a sharp decline in healthcare utilization, including cancer screening rates. The decreased cancer screening rates may lead to delayed diagnosis of early-stage cancer (49), subsequently impacting patient survival rates. Additionally, the fear of COVID-19 may deter patients from seeking medical assistance, potentially worsening their clinical outcomes (45). Therefore, it is imperative to formulate strategies to enhance cancer screening rates in response to the pandemic’s impact.

### 4.5 Comparison to other studies

According to research from the United Kingdom, palliative care faced significant challenges during the COVID-19 pandemic, necessitating immediate communication and advance care planning (ACP). Comprehensive guidelines for palliative care during emerging infectious disease outbreaks are essential (50), highlighting the importance of respecting patient autonomy and medical decision-making. Research from Brazil also indicated that stratifying and managing patients during the COVID-19 pandemic effectively identified high-risk patients for mortality (51). During the pandemic, cancer patients exhibited similar clinical presentations to non-cancer patients but had a higher risk of mortality during hospitalization (52), underscoring the importance of predicting the end-of-life for hospitalized patients. Therefore, this study analyzes the discharge planning services and palliative care needs of terminally ill hospitalized patients during the pandemic, further exploring the factors influencing high palliative care needs and mortality. This comprehensive understanding of the clinical care needs of terminally ill hospitalized patients during the pandemic enables the formulation of targeted policy recommendations, ensuring respect for patient autonomy and the provision of high-quality, comprehensive palliative care even amid the pandemic.

## 5 Limitations

This study only focused on inpatients with a palliative care needs assessment score of ≥4. Therefore, when analyzing the factors affecting high palliative care needs, data for inpatients with scores of 1 to 3 were not included. It is recommended that future studies incorporate data from inpatients with palliative care needs assessment scores of 1 to 3 and analyze the factors influencing high palliative care needs. This will result in a more comprehensive set of results. Another limitation is that the study subjects were recruited exclusively from regional teaching hospitals in Taipei City. The generalizability of the research results to other regions, especially in terms of palliative care, may vary due to differences in geographical location and healthcare systems. Patients residing in rural or underserved areas may face greater challenges, and this warrants further investigation. Although the Taiwanese government did not officially announce COVID-19 prevention measures until the end of February 2020, Taiwan’s pandemic prevention policies were gradually implemented, and the first confirmed case of COVID-19 occurred in January. The data from January and February are significant in reflecting the impact of the pandemic, and thus are included in this study. However, further research is needed to determine the representativeness of these data.

## 6 Conclusion

This study shows that palliative care faced significant challenges during the COVID-19 pandemic, placing additional pressure on medical service resources. It is recommended that terminal status be confirmed early during hospitalization, assisting patients with advance care planning (ACP) based on their or their family’s needs, and referring to interdisciplinary team members as desired. Additionally, it is crucial to enhance healthcare professionals’ awareness of death recognition, train ACP specialists, and use various tools to improve understanding and utilization of palliative care.

Furthermore, it is suggested to develop assessment tools for the preparedness of primary family caregivers for home palliative care and guidelines for palliative care during pandemics of emerging infectious diseases. This ensures that patients and their families receive adequate support and assistance during pandemic prevention periods, providing relevant care guidance to optimize the use of scarce medical resources and reduce the waste of ineffective medical resources.

## Data availability statement

The raw data supporting the conclusions of this article will be made available by the authors, without undue reservation.

## Ethics statement

The studies involving humans were approved by Institutional Review Board of Taipei City Hospital. The studies were conducted in accordance with the local legislation and institutional requirements. The participants provided their written informed consent to participate in this study.

## Author contributions

M-PW: Writing – original draft, Resources, Methodology, Investigation, Formal analysis, Data curation, Conceptualization. S-hH: Writing – review & editing, Resources, Project administration, Methodology, Investigation, Conceptualization. T-CH: Writing – review & editing, Validation, Resources, Project administration, Investigation. D-CC: Writing – review & editing, Resources, Methodology, Investigation. C-YL: Writing – review & editing, Writing – original draft, Validation, Supervision, Software, Project administration, Methodology, Investigation, Formal analysis, Data curation.
