# Supplementary material for: Impacts of COVID-19 pandemic prevention measures to the palliative care in Taiwan
Source: Front Public Health. 2024 Jul 25;12:1411185. doi: 10.3389/fpubh.2024.1411185 (PMC11309006; doi:10.3389/fpubh.2024.1411185)
Supplement: Supplementary file 1 [file Image_1.pdf]

Figure S1

Palliative Care Needs Assessment

Name: **Birth date:** **ID numer:**

Gender: **DNR: yes/no** **Date:**

A. Main diagnosis for hospitalization	2 Points /each item		B. Comorbidity (no repeated with A item)	1Point /each item	
	Nurse 1	Nurse 2		Nurse 1	Nurse 2
1. End-stage Cancer (metastasis/recurrence), give up cancer treatment.			1. Primary cancer		
2. End-stage COPD			2. Moderate COPD		
3. End-stage Liver Disease			3. Cirrhosis		
4. Kidney Dialysis (for a least 2 years & over 65 years old)			4. Other Kidney Dialysis Patient		
5. End-stage heart failure			5. Moderate heart failure		
6. Severe neurological disorders			6. Other Complex Diseases (chronic wound, catastrophic illness, multiple trauma)		
7. Other fatal acute diseases					
A total score			B total score		

C. Physical function	Nurse 1	Nurse 2
1. Completely independent, can do any work before hospitalization (0 point)		
2. Can not do rough work, can walk and do some easier work (0 point)		
3. Can not do any kind of work, can walk and self-care (1 point)		
4. Can only accomplish partial self-care on the bed or wheelchair (2 points)		
5. Completely lost function of self-care (3 points)		
C total score		

D. Other condition	1 Point /each item	
	Nurse 1	Nurse 2
1. Medical team/patient/family need help when making complex decisions and goal of care		
2. Unbearable pain		
3. Unrelievable social or spiritual concerns		
4. Repeat ED visits for the same diagnosis within 30 days		
5. Readmission for the same diagnosis within 30 days		
6. Transfer out of intensive care unit (ICU)		
7. Admitted to an intensive care unit (ICU) and have a bad prognosis		
8. Hospitalized for over 30 days		
D total score		
A+B+C+D= Palliative Care Needs Assessment Score		
Would you be surprised if the patient died within 12 months ?		
Would you consider this patient to be at the end of life?		
Do you think this patient needs palliative care?		

Signature (nurse 1)/ date: **Signature (nurse 2)/ date:**

**Doctor's opinion**	Attending Doctor.	Specialist
Would you be surprised if the patient died within 12 months ?		
Would you consider this patient to be at the end of life?		
Do you think this patient needs palliative care?		
**Signature / date:**		
